# Supplementary material for: Learning curve and surgical outcome of robotic assisted colorectal surgery with ERAS program
Source: Sci Rep. 2022 Nov 29;12:20566. doi: 10.1038/s41598-022-24665-w (PMC9709162; doi:10.1038/s41598-022-24665-w)
Supplement: Supplementary file 2 — Supplementary Information 2. [file 41598_2022_24665_MOESM2_ESM.zip › RAL Raw data-Ñ[▒K/067/HS_ñw╝╨░O▒Kñσ.pdf]

正式摘要(電子簽章：000000213343837)		
W55 / W55 ☐英文標題(注意:無英文姓名-請補病人基本檔!)	☐【讀取】憑證	

臺中榮民總醫院
出院病歷摘要

轉入號： 轉出號：

[redacted]

**(8)**流水編號	
**(9)**入院日期	110/03/24 (2021) 直腸外科 W55 - 142
**(10)**轉科日期	
**(11)**出院日期	110/03/30 (2021) 住院天數計 6 日

診斷

- 入院：Sigmoid Ca, proved at LMD
  <Hx>
  1. HTN under medication
  2. uterine myoma s/p ATH 10+ yrs ago
- 出院：Rectal Ca, proved at LMD s/p robotic assisted LAR on 2021/03/25
  <Hx>
  1. HTN under medication
  2. uterine myoma s/p ATH 10+ yrs ago

主訴

- stool OB(+) in 2021/01

病史

- This 54 y/o female with a hx of
  1. HTN under medication
  2. uterine myoma s/p ATH 10+ yrs ago
  This time, stool OB (+) was noted in regular health examination in 2021/01 at 苗栗光田. She then went to 苗栗大千 where colonscopy showed multiple sigmoid polyp with one adenocarcinoma in situ. Operation was suggested. She then came to TCVGH for second opinion. Under the impression of sigmoid colon cancer, she was admitted for Robotic AR and ERAS protocol.

體檢發現

-
  - General appearance :
    - easy looking , conscious clear , without cardiopulmonary distress
    - Vital signs自動帶入date 體溫(BT) 脈搏(P) 呼吸(R) 血壓(BP) 身高(BH) 體重(BW) BMI
      20210324 36.8℃ 72次/分 17次/分 144/86mmHg 149.4cm 57.2kg 25.6
    - Vital signs: B.P. 144/86 mmHg , P.R. 72 /min , R.R. 17 /min , B.T. 36.8 ℃
  - HEENT :
    - Eyes: no anemic conjunctiva, no icteric sclera, normal EOM
    - ENT: no otorrhea, no rhinorrhea, no erythematous mucosa, no injected tonsil, no deformity
  - Neck: supple, freely movable, trachea midline,no deformity, no engorged jugular vein , no palpable thyroid or lymph node enlargement
  - Chest:
    - Inspection: Grossly normal, symmetric and fully expansion, no accessory muscle used, no operation scar, no spider angiomata, no gynecomastia
    - Palpation: no palpable mass or nodule, no subcutaneous emphysema, no tenderness
    - Ausculation: clear breathing sound
  - Heart:
    - Palpation: no thrill, no heave
    - Percussion: normal heart size

- o Ausculation: regular heart beat, normal S1, S2, no gallop, no murmur
  - Extremities: No deformity, full ranged of motion without limitation, no clubbing finger or nail cyanosis, no pitting edema, no ulcer or pigmentation, no obvious skin lesion, no varicose vein, no bone, joint or muscle tenderness

CRS CONDITION

- Karnofsky performance status: (100) Normal, no complaints, no evidence of disease
- PE of Abdomen:
- CEA: 1.56 ng/ml
- Imaging Studies:
- Colonoscope report: s/p POLYPECTOMY--ADENOCARCINOMA IN SITU ARISING FROM TUBULLOVILLOUS ADENOMA
- Figures:
  1. colon & rectum

手術日期及方法

- 手術日期及方法20210325 Robot Assisted Colectomy(L)

住院治療經過

- After admission, robotic LAR was done smoothly on 2021/03/25. On ERAS protocol, foley was removed and she tried BLAND II diet on 03/27. Mild headache was mentioned on 03/29, symptom improved after hydration. We discharged the patient on 03/30 with stable condition.

合併症

一般檢查

- 累積報告：

DATE	TIME	ALB	TP	BIL.T	BIL.D	ALKP	AST	ALT	LDH	CHOL	TG	HDL-C	LDL	UA	NH3	AMY
1100324	2136									179	81	40		5.7		
1100325	1840	3.2		0.4	0.1	30	14	13								
1100329	0923	3.5	7.1	0.4	0.1	45	14	9	163							

DATE	TIME	WBC	RBC	HGB	HCT	MCV	PLT	BLST	NEUT	BAND	SEG	LYM	MON	EOS	BAS
1100325	1908	12290	4.16	12.3	37.3	89.7	173		92.8			6.4	0.7	0.0	0.1
1100329	0913	8970	4.56	13.6	39.9	87.5	201		83.1			12.5	3.3	0.8	0.3

DATE	TIME	NA	K	CL	CA	BUN	CREAT	Mg	P	CRP	ESR	PCT	LIPASE	LACTATE
1100325	1840	138	4.2	108	8.0									
1100329	0923	138	3.8	104	8.2	8	0.81							

DATE	TIME	GLUCOSE	PAB	TRF	ZN	CU	KETONE	CK	CKMB	PT-P	PT-C	APTT-P	APTT-C	TRO-T
1100325	1840	165												

特殊檢查

病理報告

其它

出院時情況

- 15.計劃性出院

出院指示

藥名	劑量	劑量單位	用法	頻次	數量	數量單位	現況	天數	單位
Xanax tab 0.5mg	1	TAB	PO	QNPRN	15	TAB	TH	15	天
Sevikar tab 5/20mg	1	TAB	PO	QD	15	TAB	TH	15	天
Mag. Oxide tab	1	TAB	PO	TIDPRN	45	TAB	TH	15	天
Ultracet tab	1	TAB	PO	Q6HPRN	28	TAB	TH	7	天
Panho tab 40mg	1	TAB	PO	QDAC	15	TAB	TH	15	天

出院計劃

主治醫師 2062F 蔣鋒帆 / 住院醫師 2325G 劉佳樺

正式摘要(電子簽章：**000000213343837)**

W55 / W55

doctorRank:B
